# Different Forms of TFF3 in the Human Endocervix, including a Complex with IgG Fc Binding Protein (FCGBP), and Further Aspects of the Cervico-Vaginal Innate Immune Barrier

**DOI:** 10.3390/ijms25042287

**Published:** 2024-02-14

**Authors:** Aikaterini Laskou, Eva B. Znalesniak, Sönke Harder, Hartmut Schlüter, Dörthe Jechorek, Kathrin Langer, Carina Strecker, Claudia Matthes, Svetlana N. Tchaikovski, Werner Hoffmann

**Affiliations:** 1 Institute of Molecular Biology and Medicinal Chemistry, Otto-von-Guericke University Magdeburg, Leipziger Str. 44, 39120 Magdeburg, Germany; 2 Section Mass Spectrometric Proteomics, Diagnostic Center, University Medical Center Hamburg-Eppendorf, Martinistr. 52, 20246 Hamburg, Germany; 3 Institute of Pathology, Otto-von-Guericke University Magdeburg, Leipziger Str. 44, 39120 Magdeburg, Germany; 4 Department of Gynecology and Obstetrics, Otto-von-Guericke University Magdeburg, Gerhart-Hauptmann-Str. 35, 39108 Magdeburg, Germany

**Keywords:** cervix, trefoil factor, TFF, IgG Fc binding protein, FCGBP, mucin, lectin, innate immune defense, NADPH oxidase, transgender

## Abstract

TFF3 is a typical secretory poplypeptide of mucous epithelia belonging to the trefoil factor family (TFF) of lectins. In the intestine, respiratory tract, and saliva, TFF3 mainly exists as a high-molecular-mass complex with IgG Fc binding protein (FCGBP), which is indicative of a role in mucosal innate immunity. For the first time, we identified different forms of TFF3 in the endocervix, i.e., monomeric and homodimeric TFF3, as well as a high-molecular-mass TFF3-FCGBP complex; the latter also exists in a hardly soluble form. Immunohistochemistry co-localized TFF3 and FCGBP. Expression analyses of endocervical and post-menopausal vaginal specimens revealed a lack of mucin and TFF3 transcripts in the vaginal specimens. In contrast, genes encoding other typical components of the innate immune defense were expressed in both the endocervix and vagina. Of note, FCGBP is possibly fucosylated. Endocervical specimens from transgender individuals after hormonal therapy showed diminished expression, particularly of FCGBP. Furthermore, mucus swabs from the endocervix and vagina were analyzed concerning TFF3, FCGBP, and lysozyme. It was the aim of this study to illuminate several aspects of the cervico-vaginal innate immune barrier, which is clinically relevant as bacterial and viral infections are also linked to infertility, pre-term birth and cervical cancer.

## 1. Introduction

The uterine cervical canal connects the vagina and the uterus, and thus plays a central role in fertilization. On the one hand, it protects the uterus from ascending infections; on the other hand, during pregnancy it seals the uterus via a mucus plug. The endocervix is covered by a columnar epithelium, which is the main source of mucus in the female genital tract [1]. The endocervical epithelium secretes a cocktail of polypeptides, proteins, and mucins. Its composition is under hormonal control and changes during the menstrual cycle [1,2,3,4]. Characteristic components are gel-forming mucins (MUC5AC, MUC5B, MUC6), IgG Fc binding protein (FCGBP), deleted in malignant brain tumor (DMBT1), immunoglobulins, and the trefoil factor family (TFF) peptide TFF3 [1,2,5]. The cervical mucus traps microorganisms and eliminates them via the vagina. Here, particularly mucins, FCGBP, and DMBT1 seem to play key roles. Of special note, the rheological properties of the cervical mucus change during the menstrual cycle, i.e., around the time of ovulation the mucus becomes more watery and less viscous, facilitating sperm penetration [1,6].

During pregnancy, the cervical secretions change and a cervical mucus plug is formed, sealing the nearly sterile uterus particularly from the vaginal microbiota [7]. Protein profiling points to immunological functions of the cervical mucus plug, and also identified anti-microbial factors [8,9]. Characteristic components are again the gel-forming mucins MUC5AC and MUC5B, as well as FCGBP [8]. Of note, a porous cervical mucus plug deficient in Muc5b leads to preterm birth after vaginal infection in mice [10].

The endocervical secretions are part of the innate immune defense system [1] and are comparable with those of other mucous epithelia, such as the oral cavity (salivary glands) and the intestinal tract [11,12]. The cervical mucus moves downwards and mixes with the vaginal fluid to become the cervico-vaginal mucus, which is typically less viscous than the cervical mucus. The vaginal epithelium is colonized by microbiota, with a predominance of *Lactobacillus* spp. [1]. A wide range of different mechanisms is used for protection, e.g., agglutinins (e.g., mucins, DMBT1), FCGBP, and antimicrobial peptides such as lysozyme (LYZ) and defensins [13]. Furthermore, by analogy with the intestine, the generation of extracellular reactive oxygen species (ROS), i.e., hydrogen peroxide (H_2_O_2_) and the superoxide anion radical (^•^O_2_^−^), by the NOX/DUOX family of transmembrane NADPH oxidases also play a protective role in the endocervix [14,15]. In addition, the glycosylation pattern is changed during the menstrual cycle. Before and after ovulation, sialylated oligosaccharides dominate, whereas during ovulation, fucosylation by fucosyltransferase FUT2 increases [2]. Thus, fucosylation, in particular, seems to play an important protective role due to host-microbe interactions, as already known for the intestine [16].

TFF peptides are a family of protective lectins mainly in mucus barriers [17,18]. TFF3 has been shown to be the predominant TFF peptide in the human endocervix and, to a lesser extent, in the endometrium [19]. In contrast, TFF1 and TFF2 expression was hardly detectable in the endocervix by RT-PCR analysis, and not by Western blot analysis [19]. A later study confirmed TFF3 to be the predominant TFF peptide in cervical mucus and even reported on a significant decrease after ovulation [20]. A similar change after ovulation was reported for TFF3 on transcript level for the bovine cervix [21]. There is also a report on the increase in cervical TFF3 on day 18 of the cycle when compared with days 7 and 12 [6]. The TFF3 concentration in cervical mucus plugs from women in active labor was also correlated with the viscoelastic properties of the cervical mucus plug [22].

In the past, we could demonstrate that in the human and murine intestine, human saliva, and human respiratory tract, TFF3 forms mainly a high-molecular-mass complex with FCGBP, which is indicative of a role in mucosal innate immunity [12,23,24,25]. Thus, it was one of the aims of the present study to analyze in which forms TFF3 occurs in the human endocervix and whether TFF3-FCGBP is formed. Furthermore, we compared the cervical and the vaginal mucus from the same patients at the proliferative and secretory phases of the menstrual cycle. In addition, we performed expression analysis in both the endocervix and vagina concerning selected genes encoding several components of the mucosal innate immune system. Also, rare endocervical specimens from a transgender person were included to investigate the influence of hormonal therapy. This study was designed for a better understanding of the cervico-vaginal innate immune barrier, which protects against bacterial and viral infections. This is clinically highly relevant as infections are not only linked to sexually transmitted diseases, but also to infertility, pre-term birth, and cervical cancer [1,10,26,27,28].

## 2. Results

### 2.1. Characterization of TFF3 Forms in Human Endocervix Specimens

**Figure 1:**
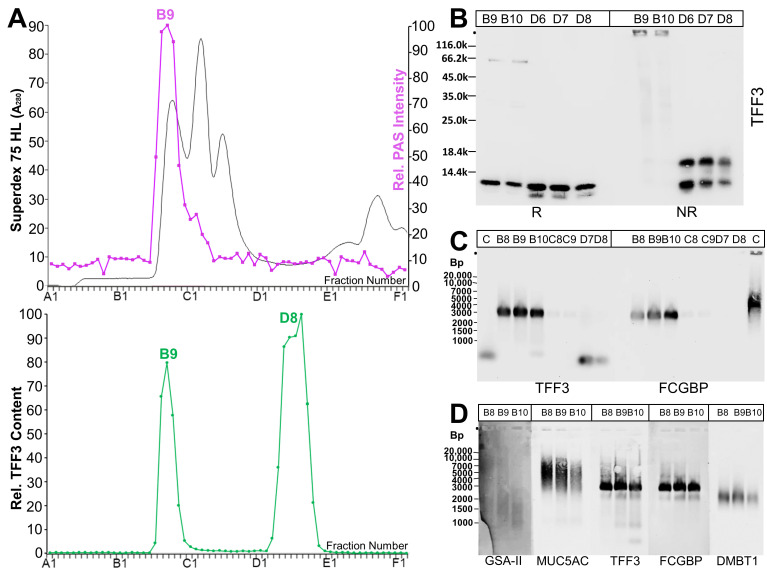
Analysis of human endocervical extract Cx-45 (E1). (**A**) Elution profile after SEC on a Superdex 75 HL column as determined via absorbance at 280 nm (PAS-positive mucin fractions: pink). Underneath: distribution of the relative TFF3 content as determined by Western blot analysis under reducing conditions and semi-quantitative analysis of monomeric band intensities. (**B**) 15% SDS-PAGE under reducing (R) and non-reducing (NR) conditions (post-in-gel reduction), respectively, and Western blot analysis of the high-molecular-mass fractions B9/B10 and the low-molecular-mass fractions D6–D8 concerning TFF3. (**C**) 1% agarose gel electrophoresis (AgGE) and Western blot analysis of the fractions B8–B10, C8/C9, and D7/D8 concerning TFF3 and FCGBP, respectively. (**D**) 1% AgGE and Western blot analysis of the high-molecular-mass fractions B8-B10 concerning MUC6 (lectin GSA-II), MUC5AC, TFF3, FCGBP, and DMBT1, respectively. Relative standard in (**C**,**D**): DNA ladder (Bp, base pairs).

As a first step, extracts from endocervix specimens (supernatants termed E1) were separated with the help of size-exclusion chromatography (SEC), and the TFF3 content was measured in each fraction. Generally, high- and low-molecular-mass forms of TFF3 were detectable (Figure 1A). The relative content of these forms varied within the five specimens investigated (Cx-25, Cx-30, Cx-32, Cx-45, Cx-50), ranging for the high-molecular-mass form from 6 to 35%. As a representative example, the results from endocervix specimen Cx-45 are shown in Figure 1. The high-molecular-mass form of TFF3 appeared together with the mucin fraction, indicated by a positive periodic acid-Schiff (PAS) reaction (Figure 1A).

Monomeric TFF3 was detectable after reduction and was completely missing under non-reducing conditions (fractions B9/B10, Figure 1B). This indicates that the high-molecular-mass form of TFF3 represents a disulfide-linked heterodimer. In contrast, the low-molecular-mass form of TFF3 (fractions D6–D8, Figure 1B) appeared as a double band under reducing conditions, indicating a somewhat shortened form. Under non-reducing conditions, two distinct bands were visible in fractions D6–D8, i.e., a monomeric form (Mr: 14k) and a form with a M_r_ of about 16k (Figure 1B).

**Figure 2:**
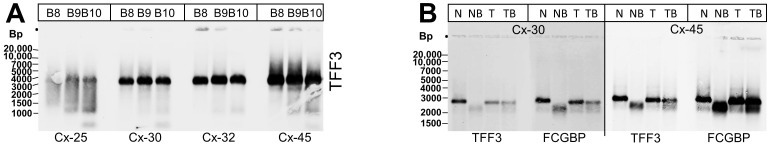
Analysis of human endocervical extracts (E1) after SEC on a Superdex 75 HL column. (**A**) 1% AgGE and Western blot analysis of the fractions B8–B10 from endocervical extracts Cx-25, Cx-30, Cx-32, and Cx-45 concerning TFF3. (**B**) 1% AgGE and Western blot analysis of fractions B9 of endocervical extracts Cx-30 and Cx-45 concerning TFF3 and FCGBP, respectively. Different treatments of the samples were compared: native fraction (N), native fraction after boiling in sample buffer (0.1% SDS) for 5 min (NB), native fraction after TRIzol extraction (T), native fraction after TRIzol extraction followed by boiling in sample buffer (0.1% SDS) for 5 min (TB). Relative standard: DNA ladder (Bp, base pairs).

As TFF3 from the human saliva [24], intestinal tract [23], and respiratory tract [25] is known to form disulfide-liked heterodimers with FCGBP, we checked after agarose gel electrophoresis (AgGE) whether TFF3-FCGBP is detectable in the high-molecular-mass fractions B8–B10 (Figure 1C). Clearly, TFF3-FCGBP was only present in the high-molecular-mass fractions, and not in the low-molecular-mass fractions D7/D8 (Figure 1C). Furthermore, the majority of TFF3-FCGBP is not associated with the mucin MUC5AC or DMBT1; only the size of the lower band overlaps with that for the DMBT1 signal (Figure 1D). TFF3-FCGBP was not only detectable in specimen Cx-45, but also in other endocervix specimens (Cx-25, Cx-30, and Cx-32; Figure 2A).

TFF3-FCGBP and FCGBP are known to form oligomers [25,29]. Thus, we tested whether endocervical TFF3-FCGBP oligomers can be dissociated by boiling and/or denaturing TRIzol extraction (Figure 2B). Clearly, boiling resulted in a shift in the TFF3-FCGBP band towards a lower M_r_ (Figure 2B/NB). In contrast, TRIzol extraction hardly showed any effect (Figure 2B/T).

**Figure 3:**
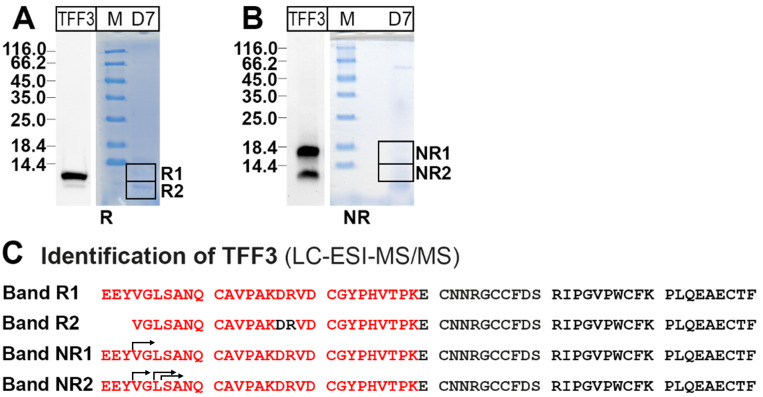
Protein analysis of the low-molecular-mass forms of TFF3 in extract Cx-45 (fraction D7 from Figure 1A). (**A**,**B**) Preparative 15% SDS-PAGE under reducing (R) and non-reducing conditions (NR). Shown are Western blot analyses concerning TFF3 and parallel Coomassie staining. Bands R1, R2, NR1, and NR2 were excised. (**C**) Results of the protein analyses after tryptic in-gel digestion of bands R1, R2, NR1, and NR2. Identified regions in TFF3 are shown in red. Figured are the longest N-terminal sequences characterized. Alternative (shortened) N-terminal sequences identified are indicated by arrows.

In order to unambiguously verify the TFF3 immunoreactive bands, particularly in the low-molecular-mass region (fraction D7; Figure 1B), the corresponding bands were eluted after reducing and non-reducing SDS-PAGE, respectively (Figure 3A,B), and TFF3 was identified by bottom-up proteomics (Figure 3C).

Of note, the N-terminal sequences of bands NR1 and NR2 showed heterogeneities (arrows in Figure 3C). Similar results were obtained after protein analysis of the low-molecular-mass forms of TFF3 after SEC (fraction D7) of extract Cx-30. TFF3 was clearly identified again.

**Figure 4:**
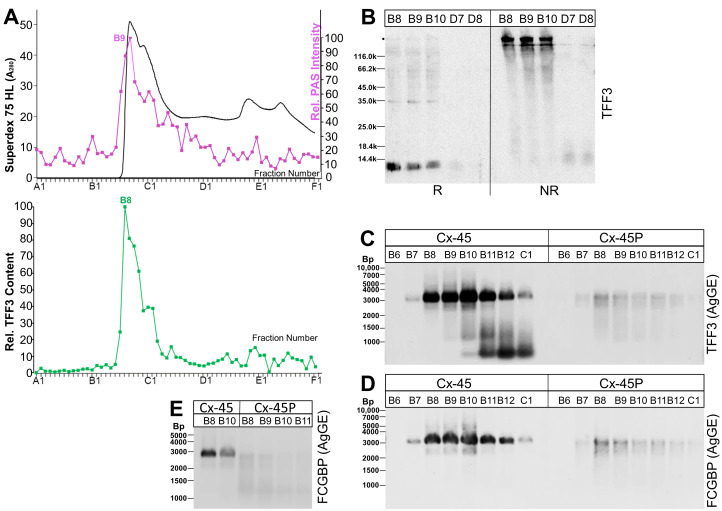
Analysis of the cell pellet from human endocervical extract Cx-45 (Cx-45P). (**A**) Elution profile of extract Cx-45P (E2) after SEC on a Superdex 75 HL column as determined via absorbance at 280 nm (PAS-positive mucin fractions: pink). Underneath: distribution of the relative TFF3 content as determined by Western blot analysis under reducing conditions and semi-quantitative analysis of monomeric band intensities. (**B**) 15% SDS-PAGE under reducing (R) and non-reducing (NR) conditions (post-in-gel reduction), respectively, and Western blot analysis of the high-molecular-mass fractions B8–B10 and the low-molecular-mass fractions D7/D8 concerning TFF3. (**C,D**) 1% AgGE and Western blot analysis of the fractions B6–C1 from SEC of the extract E1 (Cx-45, Figure 1) and the extract E2 of the cell pellet (Cx-45P, Figure 4) concerning TFF3 (**C**) and FCGBP (**D**), respectively. (**E**) Analysis of similar fractions as in (**D**) concerning FCGBP. Relative standard in (**C**–**E**): DNA ladder (Bp, base pairs).

In a next step, the remaining cell pellet from the extraction of endocervix specimen Cx-45 was extracted again in the presence of 1% SDS and the soluble constituents (termed supernatant E2) were analyzed by SEC for the presence of TFF3 (Figure 4). Here, mainly a high-molecular-mass form of TFF3 appeared (peak: B8), and nearly no low-molecular-mass forms were detected (Figure 4A). After the reduction of fractions B8–B10, monomeric TFF3 was detectable, and was missing under non-reducing conditions (Figure 4B). Instead, a high-molecular-mass band appeared (Figure 4B), which was identified as a TFF3-FCGBP complex (Figure 4C,D).

Furthermore, the high-molecular-mass fractions B6–C1 of the extract E1 (Cx-45, Figure 1) were compared with those of extract E2 (cell pellet, Cx-45P, Figure 4) concerning their TFF3 (Figure 4C) and FCGBP contents (Figure 4D). Clearly, most of the TFF3 (Figure 4C) and FCGBP immunoreactivities (Figure 4D) in the TFF3-FCGBP complex (at about 3000 Bp) appeared in the soluble extract E1 (Cx-45), about 90% (TFF3) and 86% (FCGBP), respectively. The TFF3/FCGBP ratio in the E1 extract is about 1.1, whereas in the E2 extract, it is 0.4. The hardly soluble TFF3-FCGBP complex in E2 (Cx-45P) appeared with a slightly lower M_r_ when compared with the soluble form in E1 (Cx-45; Figure 4E).

### 2.2. Transcriptional Profiling of Human Endocervical and Vaginal Specimens (RT-PCR Analyses)

Here, we investigated the expression of selected genes generally involved in the mucosal innate immune defense. On the one hand, they encode polypeptides/proteins typically involved in the formation of mucous barriers (i.e., mucins, TFF peptides, gastrokines, FCGBP, DMBT1; disulfide isomerases AGR2 and PDIA3, glycosyl transferases A4GNT and FUT2). On the other hand, genes were included encoding enzymes playing a key role for the metabolism of ROS (DUOX1, DUOX2, NOX1, NOX2, NOX5, and SOD3). In addition, the expression of lysozyme (LYZ) was analyzed. Gastrokines (GKN1 and GKN2) are polypeptides characteristic of the stomach, which are typically secreted together with MUC5AC, and GKN2 is able to form a disulfide-linked hetero-dimer with TFF1 [17,30]. AGR2 is a disulfide isomerase, which plays a major role for the folding of the mucins MUC5AC, MUC5B, and MUC6 [12,31,32,33]. TFF2 is a lectin, which binds to MUC6 via a terminal αGlcNAc residue of the carbohydrate moiety of this mucin [33]. PDIA3 is a disulfide isomerase possibly involved in the folding of several mucins. The extracellular superoxide dismutase SOD3 protects mucous epithelia by destroying extracellular superoxide, generating H_2_O_2_ [34].

Furthermore, we analyzed the expression of two genes (FCGRT, PIGR) encoding receptors responsible for the transport of immunoglobulins IgG and IgA, respectively, across epithelial cells by transcytosis, i.e., the neonatal Fc receptor (FcRn, encoded by the FCGRT gene, transport of IgG) and the polymeric immunoglobulin receptor (pIgR, encoded by the PIGR gene, transport of IgA).

**Figure 5:**
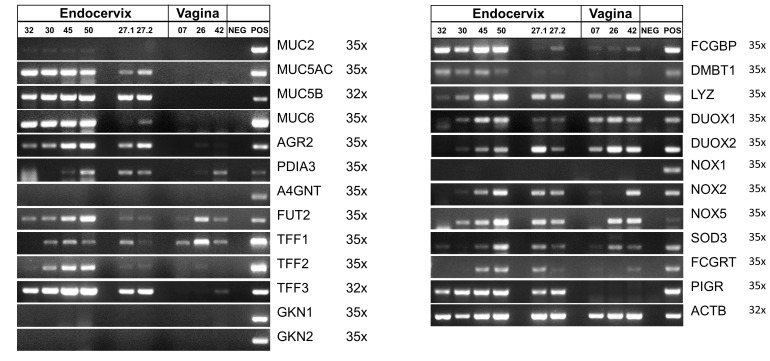
RT-PCR analyses. MUC2, MUC5AC, MUC5B, MUC6, AGR2, PDIA3, A4GNT, FUT2, TFF1-3, GKN1-2, FCGBP, DMBT1, LYZ, DUOX1-2, NOX1, 2, 5, SOD3, FCGRT, and PIGR expression was monitored in human endocervical (32, 30, 45, 50; transgender person: 27.1, 27.2) and vaginal specimens (07, 26, 42). The number of amplification cycles is given on the right. As a control, the expression of ACTB was monitored. For positive controls (POS), cDNA from stomach, duodenum, colon or bronchial BEAS-2B cells was used.

In Figure 5, the expression of the selected genes was monitored in endocervical specimens (Cx-32, Cx-30, Cx-45, Cx-50), and specimens from two different cervical areas of a transgender person after hormone therapy (Cx-27.1, Cx-27.2). For comparison, vaginal specimens were analyzed (V-07, V-26, V-42). Of note, specimens Cx-50, V-07, V-26, and V-42 were from post-menopausal patients.

Generally, most of the selected genes were expressed in the endocervical specimens. There were differences particularly concerning MUC6, FCGBP, and DMBT1 when compared with endocervical specimens from the transgender person after hormonal treatment. In the vaginal specimens, MUC5AC, MUC5B, MUC6, AGR2, TFF3, FCGBP, DMBT1, and PIGR transcripts, in particular, were lacking when compared with the endocervical specimens. MUC2 expression was hardly detectable in endocervical specimens only. A4GNT, GKN1, GKN2, and NOX1 transcripts were not detectable in both the endocervical and vaginal specimens investigated here.

### 2.3. Analysis of Endocervical Mucus Fractions concerning Fucosylation

**Figure 6:**
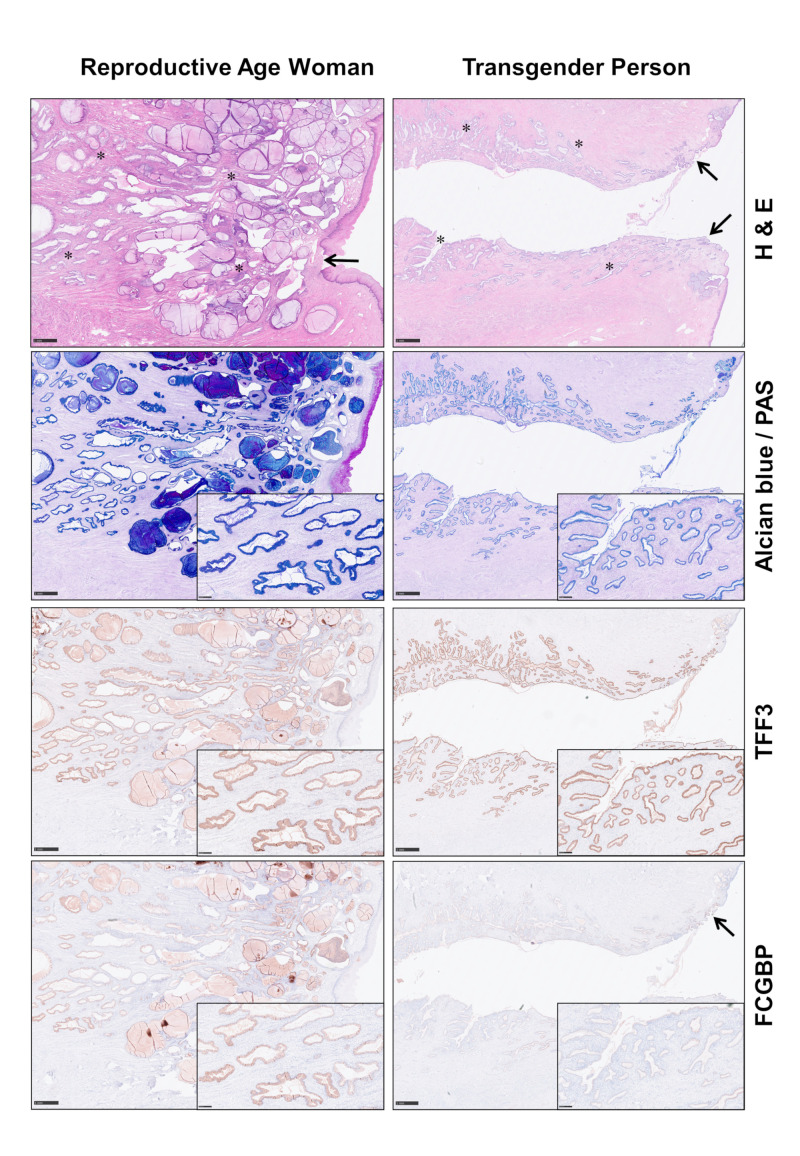
Analysis of high-molecular-mass fractions of human endocervical E1 extracts. 1% AgGE and Western blot analysis concerning fucosylation (lectin AAA) and FCGBP, respectively, of the fractions B8/B9 from three different endocervical extracts (Cx-30, Cx-32, Cx-45) after SEC (analogous samples as shown in Figure 2A). Relative standard: DNA ladder (Bp, base pairs).

As fucosyltransferase FUT2 is expressed in the endocervix (Figure 5), fucosylation was tested in high-molecular-mass fractions of endocervical extracts by the help of the lectin AAA from *Anguilla anguilla* (Figure 6), which is known for its specificity for α-L-fucose residues [35]. A faint signal was detectable co-migrating with immunoreactivity against FCGBP. This is a first indication that TFF3-FCGBP might be a target for fucosylation.

### 2.4. Analysis of Human Endocervical and Vaginal Mucus Specimens during the Menstrual Cycle (Western Blot Analyses)

**Figure 7:**
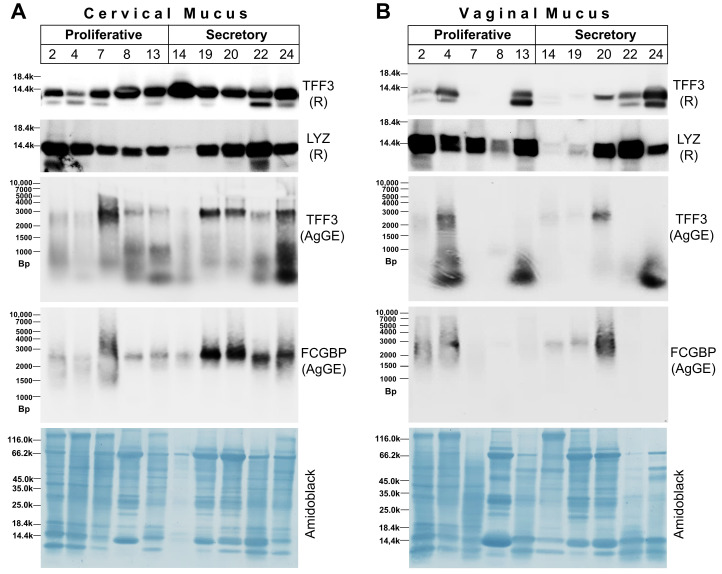
Western blot analyses of endocervical (**A**) and vaginal mucus swabs (**B**), respectively. The specimens were obtained in parallel during the proliferative (specimens 2, 4, 7, 8, and 13) or secretory phases (specimens 14, 19, 20, 22, and 24) of the menstrual cycle. (**A**) Western blot analyses of endocervical mucus specimens concerning TFF3, lysozyme (LYZ) and FCGBP, respectively, are shown after 15% SDS-PAGE under reducing conditions (R) or 1% AgGE (relative standard: DNA ladder; Bp, base pairs). As a loading control, staining with Amidoblack is represented of the same blot used for detection of TFF3 after SDS-PAGE under reducing conditions. (**B**) Analyses of vaginal mucus specimens analogous to (**A**). 15 µg protein were loaded per lane; for detection of LYZ, only 5 µg protein were loaded per lane.

In order to test whether the TFF3 and FCGBP contents show variations during the menstrual cycle, mucus swabs were taken in parallel from the endocervix and vagina, respectively, from different patients during the proliferative or secretory phases. Representative results are shown in Figure 7. The different samples contained the same amount of protein per lane as determined by the BCA protein assay kit, and Amidoblack staining of the Western blot was used as an additional loading control (Figure 7). Western blots for TFF3 after SDS-PAGE under reducing conditions are a measure for the total TFF3 content. In contrast, Western blots for TFF3 after native AgGE are a measure for both the amount of TFF3-FCGBP (high-molecular-mass range) and the low-molecular-mass forms of TFF3.

The different patients showed great individual variations in their total TFF3 content, in both the proliferative as well as the secretory phase (Figure 7). Off hand, there were no differences detectable between the proliferative and secretory phases concerning TFF3, LYZ, and FCGBP, neither in the cervical (Figure 7A) nor in the vaginal mucus (Figure 7B). TFF3 was easily detectable in all endocervical (Figure 7A) but not in all vaginal mucus specimens (Figure 7B). Generally, the total TFF3 content in the endocervical and the vaginal mucus specimens did not parallel. Of note, in the endocervical mucus specimens, the intensity of the TFF3-FCGBP signal was not parallel to that of the TFF3 signal under reducing conditions (total TFF3; Figure 7A). In the vaginal mucus, TFF3-FCGBP was hardly detectable and in the samples with the highest total TFF3 content (4, 13, 24), mainly low-molecular-mass forms of TFF3 were detectable after AgGE (Figure 7B). In contrast, the signals for lysozyme were rather comparable in the cervical and vaginal mucus specimens, with no major differences between the proliferative and secretory phases (Figure 7).

### 2.5. Localization of FCGBP and TFF3 in the Human Endocervix

**Figure 8:**
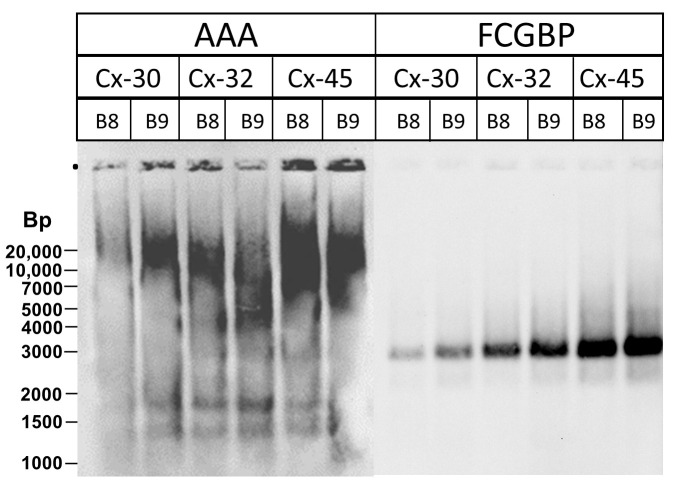
Localization of FCGBP and TFF3 in the endocervix using immunohistochemistry. Shown are serial slides of uterine surgical specimens of a woman in the reproductive age and a transgender person after hormonal therapy. Staining was with hematoxylin/eosin (HE), PAS/Alcian blue for mucus secretion, as well as an anti-TFF3 and an anti-FCGBP antiserum, respectively. Asterisks mark endocervical glands, arrows mark the squamo-columnar junction. Scale bars: 1 mm, inlay: 250 µm.

Immunohistochemistry of uterine surgical specimens showed different expression patterns for TFF3 and FCGBP in the endocervical epithelium, when women in the reproductive age (six patients) and after the menopause (six patients), respectively, were compared with transgender persons after hormonal therapy (six patients). Generally, the results obtained from women in the reproductive age and after the menopause were similar, with a tendency toward somewhat weaker expression levels in the post-menopausal group. In Figure 8, representative cases for the reproductive age group as well as the transgender group are documented. In the reproductive age woman, both TFF3 and FCGBP were clearly expressed in the columnar surface epithelium and the endocervical glands throughout the entire endocervical canal. In contrast, in the transgender person, FCGBP expression was decreased and mainly restricted to the endocervical epithelium of the squamo-columnar junction, while TFF3 expression was retained in all sections of the cervical canal. Ectocervical squamous epithelium did not show expression of TFF3 or FCGBP.

## 3. Discussion

We show, for the first time, that different TFF3 forms are typically detected in both endocervical soluble E1 extracts after SEC (Figure 1) and in endocervical mucus swabs (Figure 7A), i.e., a high-molecular-mass TFF3-FCGBP complex and low-molecular-mass forms. In contrast, in endocervical extract E2 of hardly soluble proteins, only the TFF3-FCGBP complex was traceable in significant amounts (Figure 4). Of note, TFF3 was hardly detectable after SEC in vaginal extracts and only a few vaginal mucus swabs were positive for TFF3 (Figure 7B).

### 3.1. TFF3 from the Human Endocervix Forms High-Molecular-Mass Heterodimers with FCGBP

The existence of heterodimeric TFF3-FCGBP in easily soluble endocervical E1 extracts of different patients (Figure 2A) is in agreement with previous reports on the presence of TFF3-FCGBP in the human intestine [23], saliva [24], and respiratory tract [25]. However, in the endocervix, only a minority of TFF3 exists in the high-molecular-mass TFF3-FCGBP form, i.e., 6 to 35% in the five samples analyzed. This is rather low when compared with the other organs and secretions, respectively [23,24,25].

Of note, both TFF3 and FCGBP are secreted by almost all mucous epithelia [5,36], which suggests a general biological function of the TFF3-FCGBP complex. Here, we show for the first time that TFF3 and FCGBP are co-secreted by columnar surface epithelial cells as well as endocervical glands (Figure 8). TFF3 localization in cervical epithelial cells and glands was already previously reported [19]. Co-localization of TFF3 and FCGBP was only documented in the past for human salivary glands [37].

TFF3 was easily released from FCGBP by reduction, e.g., β-mercaptoethanol (Figure 1B), but not by denaturation by boiling in 0.1% SDS or by TRIzol extraction (Figure 1B and Figure 2B). This is a strong indication that TFF3 is covalently bound to FCGBP via a disulfide bridge, as previously suggested [23]. However, it cannot, at the moment, be excluded that TFF3 binds non-covalently to the carbohydrate moiety of FCGBP by very strong lectin activity which is even resistant to boiling in SDS. This hypothesis is in line with observations indicating at least partial non-covalent binding of Tff3 and Fcgbp in the murine duodenum [12] and human colon [23]. Furthermore, a non-covalent interaction would be comparable with the non-covalent binding of the lectin TFF2 to porcine or murine gastric mucin MUC6 [38,39]. Maybe fucose residues in the carbohydrate moiety of FCGBP (Figure 6) play a role for a possible lectin binding of TFF3. Of note, boiling in 0.1% SDS changed the migration of TFF3-FCGBP somewhat during AgGE (Figure 2B), equivalent to a slight reduction in the Mr. The reason for this is not yet known, but maybe boiling in SDS changes the oligomeric structure of FCGBP. For example, TFF3-FCGBP in the human respiratory tract seems to consist of about 10 monomeric units [25].

The majority of endocervical TFF3-FCGBP does not seem to be associated with the mucin MUC5AC or DMBT1 (Figure 1D). This is comparable with the situation in the human respiratory tract [25]. Only a smaller variant of TFF3-FCGBP might interact with DMBT1 (Figure 1D). This would be in line with a previous report describing an interaction of TFF3 and DMBT1 [40]. Thus, TFF3-FCGBP seems to fulfill a function in the innate immune defense, which is not congruent to that of mucins and DMBT1 [25].

Generally, due to its IgG binding activity, FCGBP would be perfectly suited to connect the innate with adaptive immunity, particularly in the female genital tract. Here, IgG is the predominant Ig subclass and bidirectional transport across the epithelium, i.e., transcytosis, is mediated by FcRn [41,42,43]. In Figure 5, the expression of FCGRT was detectable in both the endocervix and the vagina. Its biological relevance was demonstrated by passive immunization against herpes simplex virus-2 (HSV-2), conferring protection against vaginal infection and trapping HSV-1 [43,44,45]. Also, muco-trapping of HIV-1 by antibodies against HIV-1 was reported recently [46]. Of note, the muco-trapping functions of antibodies depend on the N-glycosylation of the Fc region of IgG [47], and the FcRn is known to enhance the transcytosis of HIV-1 across epithelial cells [48]. FCGBP is a highly up-regulated defense gene after bacterial or viral infections and could influence the adherence of microorganisms as well as their clearing [11]. It is tempting to speculate that TFF3-FCGBP plays a key role in the muco-trapping, particularly of viruses, together with IgG. This has been suggested for protection against HIV [49] and SARS-CoV-2 [25,50]. For example, copy number variations in the FCGBP gene have been hypothesized as being the cause for resistance to HIV-1 infection observed in a group of women in Kenya [49].

Furthermore, we show for the first time that TFF3-FCGBP also exists in a hardly soluble form (extract E2; Figure 4). In contrast, the low-molecular-mass forms of TFF3 are hardly detectable in E2 (Figure 4A,B). This is an indication that TFF3-FCGBP might be a constituent of an insoluble matrix, which is part of the protective endocervical innate immune barrier. We expect similar situations in other mucous epithelia, such as the gastrointestinal and respiratory tracts [23,25]. The hardly soluble form of TFF3-FCGBP seems to be somewhat degraded and appeared with a lower M_r_ after AgGE when compared with the soluble form (Figure 4E). From the relative TFF3/FCGBP ratios (Figure 4C,D), one might conclude that TFF3 is enriched in the soluble TFF3-FCGBP complex in E1 when compared with the hardly soluble complex in E2.

### 3.2. Low-Molecular-Mass Forms of TFF3 in the Human Endocervix

In the samples analyzed (E1 extracts), the majority of TFF3 (65 to 94%) is present in low-molecular-mass forms. In contrast, E2 extracts (cell pellet) hardly contain any low-molecular-mass forms (Figure 4A,B). This is an indication that these TFF3 forms are easily soluble in water. In particular, monomeric and homodimeric TFF3 forms were detectable after non-reducing SDS-PAGE (Figure 1B). For identification of TFF3 in these two gel bands, a classical bottom-up proteomics approach was chosen (Figure 3C/NR1, NR2). Of note, TFF3 from both bands showed heterogeneities at the N-terminal ends, i.e., three, five or six amino acid residues were missing (Figure 3C/NR1, NR2). Currently, it is not known which proteases are responsible for the degradation. Potential candidates are kallikrein-related peptidases, which are present in human cervico-vaginal fluid and exhibit either trypsin- or chymotrypsin-like activities [3,26,51]. This could particularly explain cleavages observed after Y-3 and L-6 (Figure 3C). Furthermore, bacterial proteinases, whose lytic activities are controlled by various intrinsic inhibitors [7], could also account for the N-terminal degradation of TFF3. Of note, the N-terminal heterogeneities are different when compared with those observed in the human respiratory tract, where cleavage occurs after V-4 [25].

Furthermore, under reducing conditions, only TFF3 from the low-molecular-mass forms appeared as a double band (Figure 1B). Proteomic analysis of both bands clearly showed that the lower band represents a shortened TFF3 variant, which at least lacks the three N-terminal amino acid residues (Figure 3C/R2). Thus, it seems that binding of TFF3 to FCGBP protects from N-terminal degradation.

Currently, there are no convincing data concerning the molecular function of monomeric and homodimeric TFF3. Generally, their motogenic and anti-apoptotic effects are rather weak, which would argue against a pronounced function for mucosal restitution [17]. However, in vitro studies with cervical cancer cells reported that TFF3 overexpression correlates with increased proliferation and invasion of these cells [52]. A simple explanation could be that TFF3 acts as a lectin ligand for numerous transmembrane receptors, such as CXCR4 and CXCR7 [18,53]. As this report did not investigate FCGBP, alternative, more complex explanations are plausible. For example, FCGBP is associated with immune infiltration in glioma [54].

It is not clear how the correlation between the TFF3 concentration and the viscoelastic properties of cervical mucus plugs [22] could be explained. It is possible that the high-molecular-mass TFF3-FCGBP complex is involved (see Section 3.1). This might imply a function for TFF3 as a lectin, such as TFF1 and TFF2 [17,18]. Thus, it would be interesting to test whether *Tff3*-deficient mice [55] show pre-term birth after vaginal infection as reported for *Muc5b*-deficient mice due to a porous cervical mucus plug [10].

### 3.3. Transcriptional Profiling of the Human Endocervix and Vagina: Specific Aspects of the Vaginal Innate Immune Barrier

The major differences between the endocervical (Cx-32, Cx-30, Cx-45, and Cx-50) and the post-menopausal vaginal specimens (V-07, V-26, V-42) observed concern the expression of genes typical of mucous epithelia, i.e., MUC5AC, MUC5B, MUC6, AGR2, TFF3, FCGBP, and DMBT1, which are lacking or hardly detectable in the vagina (Figure 5). A preliminary semi-quantitative analysis of the data from Figure 5 after normalization against ACTB even revealed that the differences concerning the genes mentioned above are possibly significant. However, in order to prove statistical significance, more specimens would have to be analyzed. The expression of MUC5B, AGR2, PDIA3, FUT2, and TFF3 is in agreement with transcriptome analysis of the bovine cervix, where all these genes also show a decrease after ovulation [21]. Furthermore, the expression of MUC5AC, MUC5B, MUC6, AGR2, TFF3, FCGBP, DMBT1, and LYZ in the endocervix (Figure 5) is in agreement with a proteomic study of endocervical mucus [2]. Remarkably, the expression of the pIgR (PIGR gene), which is responsible for the active transport of IgA [43], is also lacking in the vagina. A semi-quantitative analysis revealed possible significance. The lack of IgA transport in the vagina is in agreement with previous reports [41,42]. In contrast, several other genes involved in the innate immune defense, such as FUT2, LYZ, DUOX1, DUOX2, NOX2, NOX5, and SOD3, were expressed in both the endocervix and the vagina, with great individual variations (Figure 5). Remarkably, DUOX2 even has a tendency to be higher expressed in the vagina when compared with the endocervix.

The lack of a mucous expression profile in the vagina is in line with the different cellular structures of the endocervical and the vaginal epithelium, which either consist of a columnar epithelium with mucus-secreting gland-like structures (endocervix) or represent a stratified squamous epithelium (vagina) [26,56]. Of note, only the columnar epithelium of the endocervix is joined by tight junctions excluding penetration of applied fluorescent IgG [56]. Furthermore, after the menopause, vaginal atrophy is common and a general down-regulation of cervical mucin synthesis occurs [57].

The expression of TFFs in the endocervix differs. TFF1 transcripts were hardly detectable, TFF2 transcripts were clearly traceable and TFF3 showed the highest expression (Figure 5). This is in line with a previous RT-PCR analysis and reflects previous results at the protein level [19]. In the past, TFF1 and TFF2 were not detectable on Western blots [19]. The clear expression of TFF2 is of special interest and awaits further studies at the protein level. Of note, A4GNT expression was not detectable, neither in the endocervix nor the vagina (Figure 5). Thus, the formation of a TFF2/MUC6 lectin complex via a terminal αGlcNAc residue of MUC6, which is typical of the stomach [33], cannot be expected for endocervical mucus. Furthermore, the expression of TFF1 in vaginal specimens and particularly in V-26 should be investigated at the protein level in the future.

Of note, the expression profile of the endocervical specimens of the transgender person after hormonal therapy (Cx-27.1, Cx-27.2) was different when compared with the other endocervical specimens (Figure 5). Particularly, the expression of MUC6 and FCGBP was diminished. A semi-quantitative analysis revealed possible high significance for FCGBP. Furthermore, the down-regulation of FCGBP in the transgender person was confirmed by immunohistochemistry of additional transgender patients (Figure 8). The down-regulation of MUC6 is remarkable, as the gel-forming mucins are clustered on chromosome 11p15.5 in the MUC6-MUC2-MUC5AC-MUC5B order (telomeric to centromeric) [58] but are obviously not synchronously regulated in response to testosterone therapy. Unfortunately, there are hardly any studies concerning the regulation of mucin expression by sexual hormones. However, differential regulation of MUC5AC and MUC5B expression by estradiol has been reported recently [59]. Also, the scientific literature concerning individuals taking masculinizing hormone therapy is rather sparse and mainly restricted to histologic changes [60]. For example, prostatic metaplasia was described in the cervical epithelium [60]. Although the RT-PCR results (Figure 5) were obtained from a single individual only, they are of considerable clinical interest, as we show here, for the first time, that mucin and FCGBP expression in the endocervix can be subject to differential regulations due to hormonal treatment. This might have unfavorable consequences for the mucosal innate immune defense.

The knowledge concerning the cervico-vaginal expression of the NOX/DUOX family of NADPH oxidases is rather sparse. The expression of DUOX1, DUOX2, NOX2, and NOX5 is an indication of the generation of “primary ROS”, such as H_2_O_2_ and superoxide, in both the endocervical and vaginal epithelium. DUOX1 and NOX2 were reported to exert favorable effects in cervical cancer patients [61]. The expression of SOD3 protects these epithelia by destroying extracellular superoxide and generating H_2_O_2_ [34]. Further protection from bacterial infection occurs by the secretion of lysozyme [13], which was also easily detectable at the protein level in both the endocervical and the vaginal mucus (Figure 7). Thus, both the vagina and the endocervix are possibly well protected by these different components of the innate immune defense.

Taken together, the innate immune defense of the vagina seems to be paradoxical. On the one hand, an own mucin synthesis is missing, as well as an endogenous secretion of IgA, due to the lack of PIGR expression. On the other hand, the vagina is colonized by a unique microbial community, which is often dominated by lactic acid-producing *Lactobacillus* species [57,62]. This maintains a pH below 4.5 and limits colonization by other taxa [63]. However, in a specific configuration, the vaginal microbiota frequently contains a collection of facultative and obligate anaerobes, which is associated with a vaginal pH > 4.5 [57]. Most common are candidiasis and bacterial vaginosis [62,64]. This is the reason why probiotics are widely used for vaginal health [62]. In this context, it would be interesting to test whether genetically modified *Lactobacillus* spp. secreting particularly TFF1 would be of additional benefit. For example, modified *Lactococcus lactis* strains secreting TFF peptides were effective in the prevention and healing of colitis in a mouse model [65]. Furthermore, vaginal depletion of *Lactobacillus* spp. also correlates with an increased risk of spontaneous pre-term birth [63,66]. Thus, it is not surprising that the vaginal epithelium is protected by different innate immune mechanisms, e.g., by the endogenous generation of extracellular ROS (e.g., by DUOX1, DUOX2, NOX2, NOX5; Figure 5) and secretion of antimicrobial lysozyme (Figure 5 and Figure 7). Another important component is the FcRn, which has been demonstrated to confer protective immunity to vaginal infection [43]. Of note, binding of FcRn and IgG occurs only at an acidic pH [48,67].

### 3.4. TFF3 and FCGBP Content in the Human Endocervical and Vaginal Mucus during the Proliferative and Secretory Phases of the Menstrual Cycle

In all endocervical mucus specimens (Figure 7A), TFF3 was detectable but with great individual variations. Of special note, the amounts of total TFF3 did not correlate with the amounts of TFF3-FCGBP or with the amounts of FCGBP. This indicates that the ratio of the high-molecular-mass TFF3-FCGBP and low-molecular-mass forms of TFF3 differs in the patients to great extent. These individual variations are in line with the results from SEC, which revealed a range for the high-molecular-mass form of TFF3 between 6 and 35% (Section 2.1). The relative amount of TFF3-FCGBP roughly correlates with that of FCGBP. The relative amount of lysozyme also shows individual variations and seems to be roughly inversely correlated to TFF3. However, when specimens from the proliferative phase were compared with those from the secretory phase, no convincing differences could be noticed concerning TFF3 or lysozyme. One reason might be that the samples were collected from different patients and there are already enormous individual differences between the patients. Unfortunately, it was not possible to obtain specimens from the same patients at different phases of the menstrual cycle. However, FCGBP seems to be increased in the cervical mucus during the secretory phase, which would be in line with a previous report [68].

In the vaginal mucus specimens (Figure 7B), TFF3 was clearly detectable in 60% of the samples. There were huge individual differences. There was no correlation with the amounts of TFF3-FCGBP or FCGBP or lysozyme. The amount of TFF3-FCGBP roughly correlated with that of FCGBP. The samples with the highest TFF3 concentrations (specimens 4, 13, and 24) mainly contained low-molecular-mass TFF3 (Figure 7B/TFF3/AgGE). Of note, lysozyme appeared as a double band when compared with cervical mucus. This might be an indication of the degradation of lysozyme, particularly in vaginal mucus. Again, as for cervical mucus specimens, no convincing differences were detected between the proliferative and the secretory phases of the menstrual cycle.

RT-PCR analyses of vaginal samples did not detect TFF3 transcripts (Figure 5). This seems to contrast with the results obtained from vaginal mucus specimens (Figure 7B). One explanation might be that the vaginal specimens investigated in Figure 5 were from post-menopausal patients, whereas the specimens analyzed in Figure 7B were from pre-menopausal patients. Following menopause, the estrogen level decreases, which triggers a shedding of vaginal epithelial cells leading to vaginal atrophy [26,57]. Furthermore, it cannot be excluded that the low-molecular-mass TFF3 in the vaginal mucus swabs originates from watery endocervical secretions as these TFF3 forms are easily soluble and are thus absent in E2 extracts (Figure 4A,B).

A comparison of the cervical and vaginal mucus specimens of the same patients clearly revealed that the relative concentrations of TFF3 and FCGBP do not correlate. This is an indication that TFF3 and FCGBP synthesis in the endocervix and the vagina, respectively, occurred independently and is regulated differently.

In the course of this study, we could not detect TFF3 changes at the protein level during the menstrual cycle, neither in the cervix nor in the vagina (Figure 7). The reports in the literature for cervical TFF3 expression during the cycle are contradictory [6,20,21]. However, minor amounts of TFF3 are also expressed in the endometrium [19]. Of note, transcript profiling of human endometrial specimens revealed a dramatic down-regulation of TFF3 in the secretory phase (of about 50-fold when compared with the proliferative phase) [68,69]. Furthermore, during the menstrual cycle, a transcriptional up-regulation of TFF3 expression of about 5-fold was observed between day 1 and day 4 [70]. Thus, it is tempting to speculate that endocervical TFF3 expression is also regulated during the menstrual cycle, probably with a down-regulation in the secretory phase. That would be comparable with the results obtained from the cervix of two different species on protein and on mRNA level, respectively [20,21]. However, also changes in the serum levels of TFF3 during the menstrual cycle were reported in humans, with a maximum in the late secretory phase [71]. It should be mentioned that TFF3 levels determined via an enzyme-linked immunosorbent assay (ELISA) [20,71] should be interpreted with caution as this method might not detect TFF3-FCGBP properly. Cervical TFF3 expression during the menstrual cycle still merits further investigation in the future.

## 4. Materials and Methods

### 4.1. Human Specimens

All investigations followed the declaration of Helsinki and were approved by the Ethics Committee of the Medical Faculty of the Otto-von-Guericke University Magdeburg (code: 172/21 November 2021). All patients gave written informed consent. Here, representative results are presented obtained with specimens from the endocervix from five patients (Cx-25, Cx-30, Cx-32, Cx-45, Cx-50: SEC, RT-PCR analyses), or the vagina from three patients (V-07, V-26, V-42: RT-PCR analyses), as well as mucus swabs taken in parallel from both the endocervix and vagina from ten patients during the proliferative (No. 2, 4, 7, 8, 13) or secretory phases (No. 14, 19, 20, 22, 24) of the menstrual cycle (Western blot analyses).

Surgical specimens were obtained in the course of resections with a clear clinical indication, e.g., hysterectomy. Furthermore, endocervical specimens (Cx-27) from a transgender person (transition from female to male) after hormonal therapy with testosterone were investigated. Surgical specimens were included in this study only when these were free from malignancy and not used for pathological workup. The specimens were stored at −80 °C. Mucus swabs were taken from patients presented at our outpatient department, who suffered from fertility problems or benign gynecological conditions, such as endometriosis or uterus fibroids.

In addition, we analyzed the following uterine surgical specimens by immunohistochemistry: 6 women in the reproductive age, 6 post-menopausal women, and 6 transgender persons after hormonal therapy. The specimens were obtained from hysterectomy and registered in the archives of the Institute of Pathology. Histopathological review excluded neoplastic changes.

### 4.2. Extraction of Proteins, Protein Purification by SEC

Extraction and fractionation by SEC of endocervical specimens were similarly carried out as previously described [39]. Generally, 0.6 to 1.0 g tissue was minced with a scalpel and extracted with a 10- to 15-fold amount (*w*/*v*) of buffer (30 mM NaCl, 20 mM Tris-HCl pH 7.0 plus protease inhibitors) in a Precellys^®^ 24 lyser/homogenizer as described (aqueous extracts: supernatant E1) [39]. Then, 5 mL of the extracts E1 were fractionated by SEC with the ÄKTA^TM^ FPLC system (Amersham Biosciences, Freiburg, Germany) as described (fraction numbering: A1-A12, B1-B12, etc.) using a HiLoad 16/600 Superdex 75 prep grade column (S75HL; 20 mM Tris-HCl pH 7.0, 30 mM NaCl plus protease inhibitors; flow rate: 1.0 mL/min; 2.0 mL fractions) [39]. The fractions were stored at −20 °C.

In addition, the remaining cell pellet was extracted for 3 h at 50 °C by vortexing in about the same amount of buffer (30 mM NaCl, 20 mM Tris-HCl pH 7.0 plus protease inhibitors) including 1% SDS yielding supernatant E2 after centrifugation. E2 contains hardly soluble proteins, whereas E1 contains soluble proteins.

Furthermore, mucus swabs from the endocervix and vagina were collected either with a brush (Cell Collector, servoprax GmbH, Wesel, Germany) or with a cotton swab (# 137431, CMC Medical Devices & Drugs S.L., Malaga, Spain) and stored at −80 °C. The mucus was extracted with 1 mL TRIzol^TM^ LS Reagent (ambion by life technologies, Carlsbad/CA, USA) according to the manufacturer’s protocol. The protein pellets were carefully resuspended in 100 µL to 1 mL sterile Milli-Q water containing 1% SDS, and a protease inhibitor mix at 50 °C for 6 to 10 h. After centrifugation, the protein concentration was determined in triplicate using the Pierce^TM^ BCA protein assay kit (Thermo Scientific^TM^, Rockford, IL, USA) and then stored at −20 °C. For gel electrophoresis, 15 µg protein were loaded per lane, except for the analysis of lysozyme (5 µg per lane).

### 4.3. SDS-PAGE, AgGE, and Western Blot Analysis

Denaturing vertical SDS-PAGE under reducing and non-reducing conditions, respectively, protein staining with Bio-Safe Coomassie Stain G-250 without fixation, non-denaturing horizontal AgGE, periodic acid-Schiff (PAS) staining of mucins (dot blot), and Western blot analysis (classical electrophoretic transfer after SDS-PAGE and capillary blotting after AgGE, respectively), including staining with Amidoblack, were described previously [23,25,39,72]. When indicated, gels after non-reducing SDS-PAGE were subjected to post-in-gel reduction with 1% mercaptoethanol at 50 °C for 2 min according to a previous report [39]. In addition, 1% AgGE was used to separate high-molecular-mass proteins under non-denaturing conditions [73]. As most commercial protein markers do not cover the M_r_ range above 300k, a DNA ladder was used as a relative standard for AgGE, as previously specified in detail [25].

Human TFF3 was detected with the affinity-purified polyclonal antiserum anti-hTFF3-8 against the C-terminal peptide FKPLQEAECTF of human TFF3 [23]. FCGBP was shown with PAP389Hu01 (Cloud-Clone Corp., Katy, TX, USA) against amino acids 5176-5344 of human FCGBP. Detection of the mucin MUC6 was carried out with the biotinylated lectin GSA-II from *Griffonia simplicifolia* as reported [39,74]. Fucosylation was analyzed using the biotinylated lectin AAA from *A. anguilla* (Vector Laboratories, Biozol Diagnostica Vertrieb GmbH, Eching, Germany). DMBT1 was detected with the monoclonal antibody HYB 213-6 kindly provided by Prof. U. Holmskov (University of Southern Denmark, Odense, Denmark) [75]. For the detection of the mucin MUC5AC, the polyclonal antiserum anti-hMUC5AC-2 was used [25]. Lysozyme was recognized with the polyclonal antiserum PA5-16668 (Invitrogen by Thermo Fisher Scientific Baltics UAB, Vilnius, Lithuania).

### 4.4. Identification of Proteins by Bottom-Up Proteomics

For protein identification, gel bands were excised and subjected to tryptic digestion, followed by liquid chromatography coupled to electrospray ionization and tandem mass spectrometry (LC-ESI-MS/MS) [25]. The data obtained were processed and analyzed as described [25].

### 4.5. RNA Extraction, PCR Analysis

Isolation and purification of total endocervical and vaginal RNA, respectively, (TRIzol^TM^ Reagent; ambion by life technologies, Carlsbad, CA, USA) including digestion with RNAse-free DNAse I (Thermo Scientific, Schwerte, Germany), as well as RT-PCR (reverse transcriptase: Takara Bio Europe, Saint Germain en Laye, France) were as described [39]. The concentration and purity of the RNA were estimated with a Nanodrop ND-1000 spectrophotometer (Thermo Scientific, Peqlab Biotechnologie GmbH, Erlangen, Germany). RNA was stored at −80 °C, cDNA at −20 °C.

The specific primer pairs used for RT-PCR have been published previously (TFF1, MD11/MD12; TFF3, MD9/MD10) [76] or are listed in Table 1 (ACTB, AGR2, A4GNT, DMBT1, DUOX1, DUOX2, FCGBP, FUT2, GKN1, GKN2, LYZ, MUC2, MUC5AC, MUC5B, MUC6, NOX1, NOX2, NOX5, PDIA3, SOD3, TFF2). All primer pairs are intron spanning.

### 4.6. Immunohistochemistry

Formalin-fixed, paraffin-embedded serial tissue sections (3 µm) were dewaxed in xylol and rehydrated by descending concentrations of ethanol. For each specimen standard, hematoxylin and eosin (HE) staining, Alcian blue/PAS-staining and immunohistochemistry were performed. For antigen detection, we used the automated immunohistochemistry slide staining system VENTANA BenchMark ULTRA (Roche Diagnostics GmbH, Mannheim, Germany), the VENTANA iVIEW DAB Detection Kit (Roche Diagnostics GmbH) and the indirect biotin-streptavidin method before counterstaining with haemalaun solution. Antigen retrieval was performed with CC1mild (pH 8.5, 36 min, 95 °C) or CC2mild (pH 6.0, 44 min, 91 °C), respectively, followed by incubation with specific primary antibodies recognizing FCGBP (see Section 4.3) or TFF3 (see Section 4.3), at 36 °C for 32 min, dilution 1:500.

## 5. Conclusions

In the course of this study, monomeric and homo-dimeric TFF3 forms were characterized in the endocervix, as well as a high-molecular-mass complex with FCGBP. Part of the latter also exists in a hardly soluble form. For the TFF3-FCGBP complex, a role in mucosal innate immune defense is expected, e.g., a function for the clearing of microorganisms and as a trap for viral-antibody complexes (muco-trapping). Furthermore, we demonstrated the expression of genes in the endocervix and vagina encoding additional important components of the innate immune defense. The data presented are of clinical relevance as bacterial and viral infections are not only linked to sexually transmitted diseases, but also to infertility, pre-term birth, and cervical cancer.

## Figures and Tables

**Table 1:** Oligonucleotides used for RT-PCR analysis and calculated size of the products.

**Genes** **Accession No.**	**Primer** **No.**	**Primer Pairs**	**Nucleotide** **Positions**	**Annealing T** **Size (bp)**
ACTB NM_001101.5	MB2931 MB2932	GGATTCCTATGTGGGCGACGA GCGTACAGGGATAGCACAGC	234–254 515–496	60 °C 282
AGR2 NM_006408.4	MB3019 MB3020	AAGGCAGGTGGGTGAGGAAA AGGACAAACTGCTCTGCCAA	41–60 389–370	60 °C 349
A4GNT NM_016161.3	MB3009 MB3010	CCGATGCCCTCAAACTCCAC ATTCCCACAAAAAGGGGTGGT	488–507 824–804	60 °C 337
DMBT1 NM_004406.3	MB3015 MB3016	TGCGCTGCTCAGGCTA TGATGGTCGGCAATGTGTCT	1321–1306 1455–1436	60 °C 150
DUOX1 NM_175940.3	MB3033 MB3034	ACTTCTGGTTGGGGCATGGA TTGCTAAGGTCTCGGGGGTT	184–203 393–374	60 °C 210
DUOX2 NM_014080.4	MB1577 MB1578	GATGGTGACCGCTACTGGTT GCCACCACTCCAGAGAGAAG	1751–1770 2073–2054	60 °C 323
FCGBP NM_003890.2	MB2923 MB2924	CCTACAGCCACTCTGTGTCG TCCAGCTACTTGCGAACTCC	1612–1631 1929–1910	60 °C 318
FCGRT NM_001136019.3	MB3351 MB3352	CTCTCCCTCCTGTACCACCTTACC ATAGCAGGAAGGTGAGCTCCTTGT	186–209 642–619	60 °C 457
FUT2 NM_000511.5	MB1994 MB1995	CACTGAGGTGCCTGCCCAACC GCAGCACCGGCAGGGTGATT	58–78 464–445	60 °C 407
GKN1NM_019617.3	MB2392 MB2393	CCTCTGTCCACTGCTTTCGT CTGGTTGCAGCAAAGCCATT	77–96 326–307	60 °C 250
GKN2 NM_182536.2	MB2264 MB2265	ATCCACATCTTCAAGCCCATA CAACCACTTCCCCCTTATACA	27–47 572–552	60 °C 546
LYZ NM_000239.3	MB3021 MB3022	GGGGAATCAGCCTAGCAAACT GGATCACGGACAACCCTCTTT	144–164 390–370	60 °C 247
MUC2 NM_002457.2	MB2260 MB2261	CTGAGGGCACCATGAACTAC GGGCCGTTTGATGATACAGT	14,439–14,420 15,027–15,008	60 °C 608
MUC5AC NM_001304359.2	MB2929 MB2930	TGCCCCAACATCAGGAACAG AGTGGTCATAGGCTTCGTGC	1863–1882 2156–2137	60 °C 294
MUC5B NM_002458.3	MB326 MB327	CTGCGAGACCGAGGTCAACATC TGGGCAGCAGGAGCACGGAG	17,071–17,092 17,485–17,466	60 °C 415
MUC6 NM_005961.3	MB2927 MB2928	CACCCGAGTTCCCACATCAG CATGCACCCCTTGAACGTGA	6894–6913 7155–7136	60 °C 262
NOX1 NM_007052.5	MB2885 MB2886	GCTCCAAACCACCTCTTGAC CAGATTGCGACACACAGGAAG	200–219 445–425	60 °C 246
CYBB(NOX2) NM_000397.4	MB3023 MB3024	TTCTGGTTTGGCTGGGGTTG TCGGGCATTCACACACCATT	63–82 409–390	60 °C 347
NOX5 NM_024505.4	MB3025 MB3026	CCCTGAAGGCTGTAGAGGCA CATGGATGAGCAGGGTCAGT	74–93 315–296	60 °C 242
PDIA3 NM_005313.5	MB3027 MB3028	GCAAGCAGCGGGTTAGT ACAGGTGTTAGTGTTGGCAGT	13–29 378–358	60 °C 366
PIGR NM_002644.4	MB3355 MB3356	GCCAATGACAACATGGGAGC GATTGTCATGGGTGCAGGGA	2264–2283 2516–2497	60 °C 253
SOD3 NM_003102.4	MB2863 MB2864	GGTGCAGCTCTCTTTTCAGGA ATCTCCGTGACCTTGGCGTA	30–50 229–210	60 °C 200
TFF2 NM_005423.4	MB2228 MB2229	ATAACAGGACGAACTGCGG ATGAAGCTGATAAGGCGAAGT	252–270 612–592	60 °C 361

## Data Availability

The original contributions presented in the study are included in the article, further inquiries can be directed to the corresponding author/s.

## Abbreviations

AgGE	Agarose gel electrophoresis
FCGBP	IgG Fc binding protein
FUT	Fucosyltransferase
LYZ	Lysozyme
PAS	Periodic acid-Schiff
SEC	Size exclusion chromatography
SDS-PAGE	Sodium dodecyl sulfate–polyacrylamide gel electrophoresis
TFF	Trefoil factor family
